# *Ncf1* Governs Immune Niches in the Lung to Mediate Pulmonary Inflammation in Mice

**DOI:** 10.3389/fimmu.2021.783944

**Published:** 2021-12-14

**Authors:** Mengyao Li, Wentao Zhang, Jing Zhang, Xiaowei Li, Fujun Zhang, Wenhua Zhu, Liesu Meng, Rikard Holmdahl, Shemin Lu

**Affiliations:** ^1^ Department of Biochemistry and Molecular Biology, Institute of Molecular and Translational Medicine, School of Basic Medical Sciences, Xi’an Jiaotong University Health Science Center, Xi’an, China; ^2^ National Joint Engineering Research Center of Biodiagnostics and Biotherapy, Second Affiliated Hospital, Xi’an Jiaotong University, Xi’an, China; ^3^ Key Laboratory of Environment and Genes Related to Diseases (Xi’an Jiaotong University), Ministry of Education, Xi’an, China; ^4^ Section for Medical Inflammation Research, Department of Medical Biochemistry and Biophysics, Karolinska Institute, Stockholm, Sweden

**Keywords:** asthma, macrophages, Th1 response, ILC2, Ncf1

## Abstract

Neutrophil cytosolic factor 1 (*Ncf1*) is a major genetic factor associated with autoimmune diseases and has been identified as a key player in autoimmune mediated inflammation. We addressed the role of *Ncf1* in an antigen-induced pulmonary inflammation model, and found that the *Ncf1^m1j^* mutation, causing a deficient reactive oxygen species response, alleviated disease. The *Ncf1^m1j^* mutation was associated with a reduced inflammatory cell infiltration in airways, but had limited effect on mucus secretion, antibody production and lung fibrosis. The disease remission in the *Ncf1* mutated mice was reversed when functional *Ncf1* was transgenically expressed in alveolar macrophages, suggesting that the cellular inflammation was depended on functional *Ncf1* in alveolar macrophages. By determining cytokine and chemokine profiles in lung and serum, we found that *Ncf1* deficiency allowed an increased expression of Th1 cytokines, including TNF-α, IFN-γ and IL-12. Since also epithelial cytokines were found to be regulated by *Ncf1*, we tested the effect of *Ncf1* in IL-33 and IL-25 induced lung inflammation models. Mice with the *Ncf1^m1j^* mutation showed less sensitivity to IL-33, but not IL-25, induced lung inflammation, in a macrophage independent manner. The mice with deficient *Ncf1* showed a reduced eosinophil infiltration and group 2 innate lymphoid cell (ILC2) activation. The production of IFN-γ in CD4^+^ T cells was increased, whereas IL-5 and IL-13 in ILC2 were decreased. Importantly, anti-IFN-γ antibody treatment of *Ncf1* deficient mice increased eosinophil infiltration and rescued ILC2 activation in the lung. We conclude that *Ncf1* deficiency enhances Th1 response, deactivates ILC2, and protects against pulmonitis.

## Introduction

Allergic asthma is characterized by airway inflammation, smooth muscle contraction, epithelial cell exfoliation, over secretion of mucus and bronchial hyperresponsiveness, and is a common complex disease associated with a combination of genetic and environmental factors (1). It is known that environmental allergens, viruses, bacteria and smoking can irritate the airway epithelium, activating exaggerated innate and adaptive immune responses, and triggering the development of asthma (2). The enhanced T helper (Th) 2 immune response mediates the allergic inflammation, which is characterized by the production of interleukin (IL)-4, IL-5 and IL-13, and further results in eosinophil infiltration, mucus secretion, and airway remodelling (3). Interestingly, activation of group 2 innate lymphoid cells (ILC2) seems to precede a Th2 response. The ILC2 are activated by the airway epithelial-derived cytokines including IL-25, IL-33 and thymic stromal lymphopoietin (TSLP), leading to the production of the Th2 cytokines IL-5 and IL-13, and also activation of eosinophils (2, 4, 5). In addition, Th17 cytokines could synergize with Th2 cytokines in the pathogenesis of asthma (6, 7). Although Th2 mediated immunity seems to be involved in asthmatic diseases, it is also important in the physiology and its precise role in the pathogenicity is more difficult to understand. For this it would be helpful to unravel the role of the disease-causing genes and environmental factors.

Many chromosomal loci have been reported to be associated with asthma susceptibility, but none has so far been conclusively identified and validated functionally (8). To position genetic polymorphisms associated with complex diseases is a challenging task but more recently several single nucleotide polymorphisms (SNPs) associated with chronic inflammation have been identified in animal models (9–11). The first finding was the positionally cloning of the neutrophil cytosolic factor 1 (*Ncf1*) gene to be associated with arthritis in a rat model (12). Subsequently, it was confirmed that *Ncf1* is also associated with a variety of chronic inflammatory diseases in both animals but importantly also in humans, such as rheumatoid arthritis and systemic lupus erythematosus (SLE) (13, 14). The Ncf1 protein (earlier denoted p47phox) is a critical component of the NADPH oxidase (NOX) 2 complex, mediating the induction of reactive oxygen species (ROS) by inflammatory stimuli. Only a few studies have investigated the role of *Ncf1* in lung diseases. We have found that *Ncf1* mutant mice lacking ROS are less susceptible to transplanted lung tumors (15). A longer progression free survival (PFS) of patients with primary non-small cell lung cancer (NSCLC) was associated with higher levels of *Ncf1*, and low levels of *Ncf1* may be used as a biomarker for predicting response of patients with primary NSCLC to anti-PD-1 therapy (16). A fully functional *Ncf1* gene was found to be required for development of isocyanate-induced lung inflammation (17), and for development of SARS and influenza virus induced lung hyperinflammation in the mouse (18). However, the role of *Ncf1* in the pulmonary inflammatory diseases like asthma remains unknown.

In the present study, we have investigated the immune regulatory role of *Ncf1* in asthma, and found that mice with the *Ncf1^m1j^* mutation, with a dysfunctional ROS response, were protected from ovalbumin (OVA)-induced lung inflammation in an alveolar macrophage dependent manner, with reduced inflammatory cell infiltration in airways and an increased Th1 cytokine expression in the lungs. Interestingly, the *Ncf1* deficiency was associated with a reduced ILC2 activation, which alleviated lung inflammation caused by IL-33. Our study reveals a new candidate involved in skewing the Th1/Th2 balance, which provides a new insight in understanding the pathogenesis of asthma, and also a potential target for the disease treatment.

## Materials and Methods

### Mice

B10Q mice (C57/Bl10.Q/rhd) were originally from Jan Klein (Tübingen University, Tübingen, Germany) (19), and maintained in the medical inflammation research lab led by Rikard Holmdahl (rhd). B10Q.*Ncf1* ^*^ mice indicate B10Q mice with a mutation in the *Ncf1* gene (m1j), in which the NOX2 complex function is impaired but not deleted (19, 20). B10Q.*Ncf1* ^*^.MN strain transgenically expresses *Ncf1* under the control of the CD68 promotor in a B10Q mouse having the *Ncf1* ^m1j^ mutation, thus expressing *Ncf1* in macrophages only. The above mice were maintained in SPF animal feeding equipment of the Experimental Animal Center of Xi’an Jiaotong University Health Science Center. In this study, female mice aged 8 to 12 weeks were used. All animal experiments followed ARRIVE guidelines and were approved by the Institutional Animal Ethics Committee of Xi’an Jiaotong University.

### Antigen-induced Pulmonary Inflammation (AIPI)

Female B10Q, B10Q.*Ncf1* ^*^ and B10Q.*Ncf1* ^*^.MN mice were used to induce AIPI model essentially following previously described protocols (10). A colloid emulsion was prepared by mixing 0.1 mg/ml OVA (Sigma) and Imject™ Alum (Pierce, Thermo Fisher Scientific) in equal volume. The mice were sensitized by intraperitoneal injection of the OVA emulsion (200 μl per mouse) at days 0 and 7. From day 14, the mice were challenged with 1% OVA aerosol for 30 min per day for 7 consecutive days. On day 21, blood, bronchoalveolar lavage fluid (BALF) and lung tissues were collected.

### Histological Staining

Lung tissues from the lower left lobe were fixed with 4% paraformaldehyde (SinoPharm, China), embedded in paraffin (Hualing, Shanghai, China), sectioned, and then stained with hematoxylin and eosin (H&E, Solarbio, China), periodic acid Schiff (PAS, Solarbio, China) and Masson (Solarbio, China) (10). Peritracheal inflammatory cell infiltration was scored by an investigator with no knowledge of the sample identities, according to the number of layers of infiltrating cells, as previously described (10). Score 0: no peritracheal infiltrating cells. Score 1: sporadic infiltrating cells around the tube, less than one layer; Score 2: peritubule infiltrating cells reached 1-2 layers; Score 3: peritubule infiltrating cells up to 2-3 layers; Score 4: peritubule infiltrating cells with 3-5 layers; Score 5: more than 5 layers with peritubule infiltrating cells. Both the portion of goblet cell staining of airway mucus and the degree of airway fibrosis were scanned using Image Pro Plus software and normalized with relative airway area.

### Enzyme Linked Immunosorbent Assay (ELISA)

To quantify the levels of OVA-specific IgE and OVA-specific IgG1 in serum, a 96-well plate was coated with OVA (5 μg/ml, Sigma) overnight and blocked using 1% bovine serum albumin (BSA, Amresco). Diluted sera were added for 2 hour incubation, followed by rat anti-mouse IgE-biotin (clone No. 23G3, SouthernBiotech) or rat anti-mouse IgG1-biotin (clone No. LO-MG1-2, Bio-Rad) antibodies, and developed with streptavidin-horseradish peroxidase (HRP, R&D systems, Bio-techne). The reaction was visualized by using the substrate ABTS (Roche) and read at 405 nm using a microplate reader (Thermo Electron Corporation) (10).

### Luminex Assay

Selected cytokines and chemokines including chemokine (C-X-C motif) ligand (CXCL) 1 (range: 9.9-7220 pg/ml), tumor necrosis factor (TNF)-α (0.81-590 pg/ml), IL-12p70 (14.95-10900 pg/ml), IL-1β (76.74-55940 pg/ml), vascular endothelial growth factor (VEGF, 1.48-1080 pg/ml), IL-4 (20.44-14900 pg/ml), IL-5 (1.62-1180 pg/ml), IL-6 (8.41-6130 pg/ml), IL-10 (4.6-3350 pg/ml), IL-13 (16.69-12170 pg/ml), IL-17 (14.12-10290 pg/ml), interferon (IFN)-γ (4.39-3200 pg/ml), IL-33 (27.59-20110 pg/ml), IL-25 (46.82-34130 pg/ml), C-C motif chemokine ligand (CCL) 11 (4.24-3090 pg/ml) were quantified in serum samples of mice using a 15-plex Luminex panel (LXSAMSM-15, R&D Systems). The sample was diluted twice and the plate was detected by Luminex X-200.

### mRNA Quantification

Total RNA was extracted from lung tissue of mice using TRIzol reagent (Invitrogen) and cDNA was synthesized using the first strand cDNA synthesis Kit (Fermentas). Agilent Stratagene Mx3005P performed real-time quantitative polymerase chain reaction (RT-qPCR) with FastStart Universal SYBR Green Master (Roche) to assess cytokine expression. Mouse *β-actin* gene was used as a housekeeping gene. The primers used in this experiment are listed in the **Supplementary Table 1**.

### Epithelial Cytokine Induced Lung Inflammation

On day 0 and day 1, female mice with three genotypes were treated with IL-33 (0.5 μg/mouse, SinoBiological) (21) or IL-25 (1.5 μg/mouse, SinoBiological) (22) intranasally, respectively. On the 3rd day, mice were sacrificed and the whole lungs were taken and digested for flow cytometry assay.

### Flow Cytometry

Lung tissues were minced and digested by using collagenase type I (1 mg/ml, Sigma) and DNase (25 μg/ml, Sigma) for 1 h. Cells from lung tissue or BALF were first stained with LIVE/DEAD Fixable Violet stain kit (Thermo Fisher), and labeled with the following monoclonal antibodies for the membrane antigen staining: anti-CD45.2-BV785, anti-CD11b-APC (allophycocyanin), anti-CD11c-APC-Cy7, anti-F4/80-FITC, anti-Ly6G-Pacific blue, anti-Siglec F-PerCP (peridinin chlorophyll protein)-Cy5.5, anti-γδ T cell receptor (TCR)-PE (phycoerythrin), anti-TCRβ-Alexa Fluor 700, anti-B220-PE-Cy7, anti-CD4-PE-Cy5, mouse lineage antibody cocktail-PerCP-Cy5.5, anti-ST2-BV421 and anti-CD68-Pacific blue. For cytokine staining, cells were pre-treated with phorbol 12-myristate 13-acetate (PMA, 100 ng/ml, Sigma), ionomycin (1 μg/ml, Sigma) and brefeldin A (10 μg/ml, Selleck) for 3 h, fixed and permeated by using BD Cytofix/Cytoperm after membrane staining, and labeled with the following monoclonal antibodies for intracellular staining: anti-IL-5-PE, anti-IL-13-PE-Cy7, anti-IFN-γ-Pacific blue. Mouse lineage antibody cocktail could react with cells from the major hematopoietic lineages, such as T cells, B cells, monocytes/macrophages, NK cells, erythrocytes, and granulocytes, which includes clone 145-2C11 (recognizes CD3e), M1/70 (recognizes CD11b), RA3-6B2 (recognizes B220), TER-119 (recognizes Ly-76), and RB6-8C5 (recognizes Ly-6G and Ly-6C). The detailed antibody information is shown in **Supplementary Table 2**. Samples were analyzed using an Agilent NovoCyte 3005 flow cytometer. Immune cell gating strategy and the fluorescence-minus-one (FMO) staining controls are shown in **Supplementary Figures 1, 2**.

### Anti-IFN-γ Antibody Intervention

On day 0, IFN-γ antibody or isotype IgG (200 μg per mouse, Bio X Cell) was injected intravenously to B10Q.Ncf1^*^ mice. Then on day 0 and day 1, mice were treated with IL-33 (0.5 μg per mouse, SinoBiological) intranasally. On the third day, the whole lung was isolated, and flow cytometry was performed after digestion.

### Statistical Analysis

Graphpad Prism software was used for statistical analysis and graph construction. The quantitative data are expressed by mean ± SEM. The differences were statistically analyzed using one‐way ANOVA with post‐hoc comparison (Fisher’s) test among three experimental groups or the student’s *t* test between two experimental groups. A *P* value of less than 0.05 is considered significant.

## Results

### Residential Macrophage-Specific *Ncf1* Contributes to Inflammatory Cell Infiltration to Mediate AIPI in Mice

**Figure 1:**
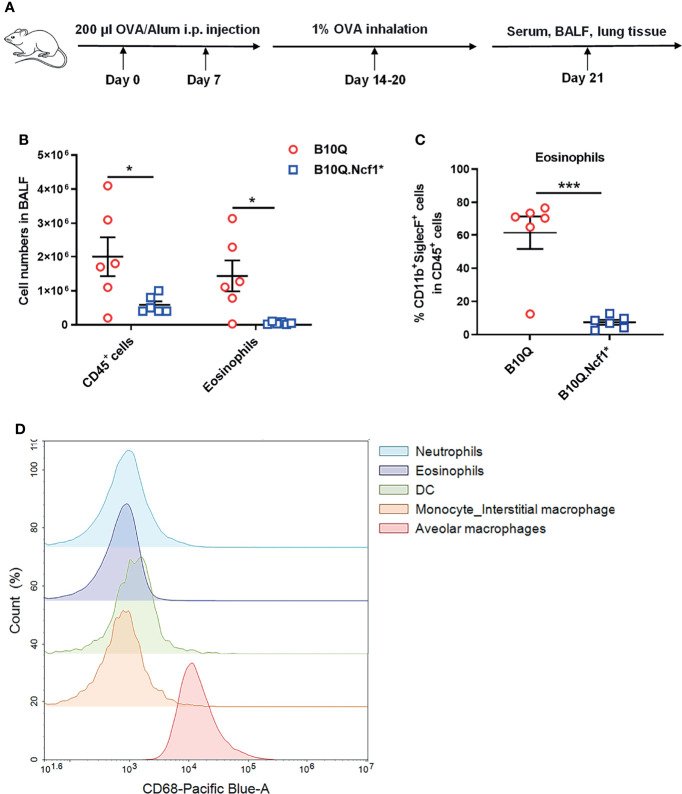
Systemic deficiency of Ncf1 alleviates lung inflammation in mice. **(A)** A scheme of antigen-induced pulmonary inflammation model in mice. B10Q and B10Q.*Ncf1* ^*^ mice were induced to develop AIPI (n = 6 per group). **(B, C)** The number of CD45^+^ cells and eosinophils in BALF were analyzed by flow cytometry, and the proportion of eosinophils in CD45^+^ cells was determined. **(D)** The CD68^+^ cells in BALF were also identified by flow cytometry. All values are expressed as means ± SEM. *P < 0.05 and ***P < 0.001, using student’s *t* test.

To determine the role of *Ncf1* in lung inflammation, B10Q.*Ncf1* ^*^ mice were compared with B10Q littermates. We did not find any significant difference in lung tissue structure, or change in the composition of immune cell populations in naïve mice (**Figure S3A–C**). Then we investigated the AIPI model using OVA as an antigen, to induce symptoms resembling asthma (**Figure 1A**). We found that the total number of immune cells in the BALF were lower in B10Q.*Ncf1* ^*^ mice (**Figure 1B**). Particularly, the number and the cell proportion of eosinophils were both reduced in B10Q.*Ncf1* ^*^ mice compared with the wild-type littermates (**Figures 1B, C**). Thus, a functional *Ncf1* was important to allow an allergic inflammation in the lung.

*Ncf1* is highly expressed in phagocytic cells, including macrophages and neutrophils (23). We next addressed the role of *Ncf1* in macrophages, due to their central regulatory role in both innate and adaptive immunity. A transgenic mouse strain, B10Q.*Ncf1* ^*^.MN (MN) was used, which transgenically expresses physiologic levels of functional *Ncf1* with the help of a CD68 promotor (24). We confirmed CD68 expression in BALF cells by flow cytometry and found that it was highly expressed on residential alveolar macrophages in lung, but not on infiltrating interstitial macrophages, neutrophils, eosinophils or dendritic cells (DC), suggesting that alveolar macrophages of B10Q.*Ncf1* ^*^.MN mice had functional *Ncf1* (**Figure 1D**).

**Figure 2:**
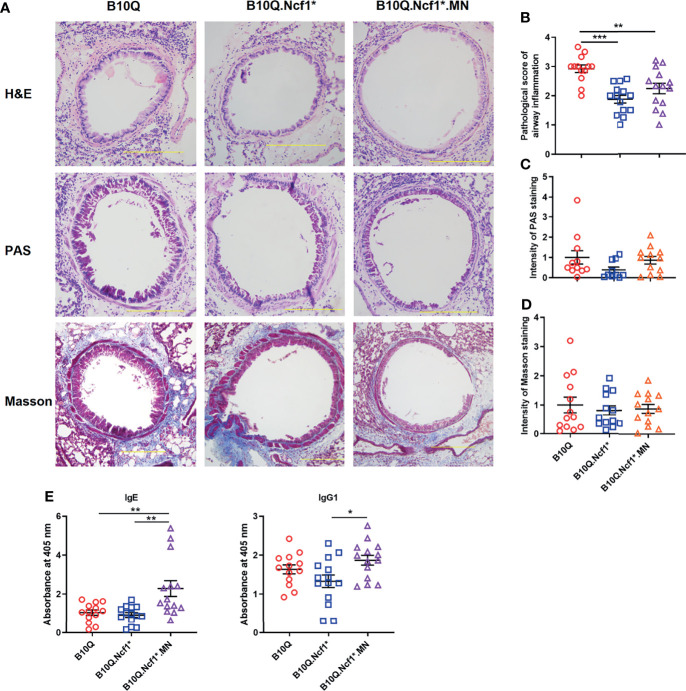
Residential macrophage specific Ncf1 contributes to the development of AIPI in mice. **(A)** Lung tissues of AIPI model induced in B10Q, B10Q.*Ncf1* ^*^ and B10Q.*Ncf1* ^*^.MN mice were stained by H&E, PAS and Masson staining (n = 10-14 per group). Bar=200 μm. **(B)** Assess the degree of inflammatory infiltration around the airways. **(C, D)** Assess the airway mucus generation and the degree of airway fibrosis with Image Pro Plus software. **(E)** The concentrations of OVA-specific IgE and IgG1 in serum were detected by ELISA, and the relative values are shown as the absorbance at 405 nm (n = 13-14 per group). All values are expressed as means ± SEM. *P < 0.05, **P < 0.01 and ***P < 0.001, using one‐way ANOVA with post‐hoc comparison (Fisher’s) test.

We then induced AIPI in the three strains, wild-type, *Ncf1* ^*^ and MN mice. Histological analyses were performed on lung tissues showing the extent of inflammatory infiltration around the airway, mucus secretion of airway epithelia and airway fibrosis (**Figure 2A**). H&E staining showed that the degree of peri-airway inflammatory infiltration in *Ncf1* deficient mice was significantly lower than that in wild-type mice, and MN tended to compensate for this difference (**Figure 2B**). However, *Ncf1* deficiency had no significant effect on the production of airway mucus and the airway fibrosis, although a trend in the PAS staining could be observed (**Figures 2C, D**). At the same time, we found that *Ncf1* deficient mice showed no difference in the production of OVA-specific IgE and IgG1 compared with wild-type, although MN mice showed higher antibody levels (**Figure 2E**).

**Figure 3:**
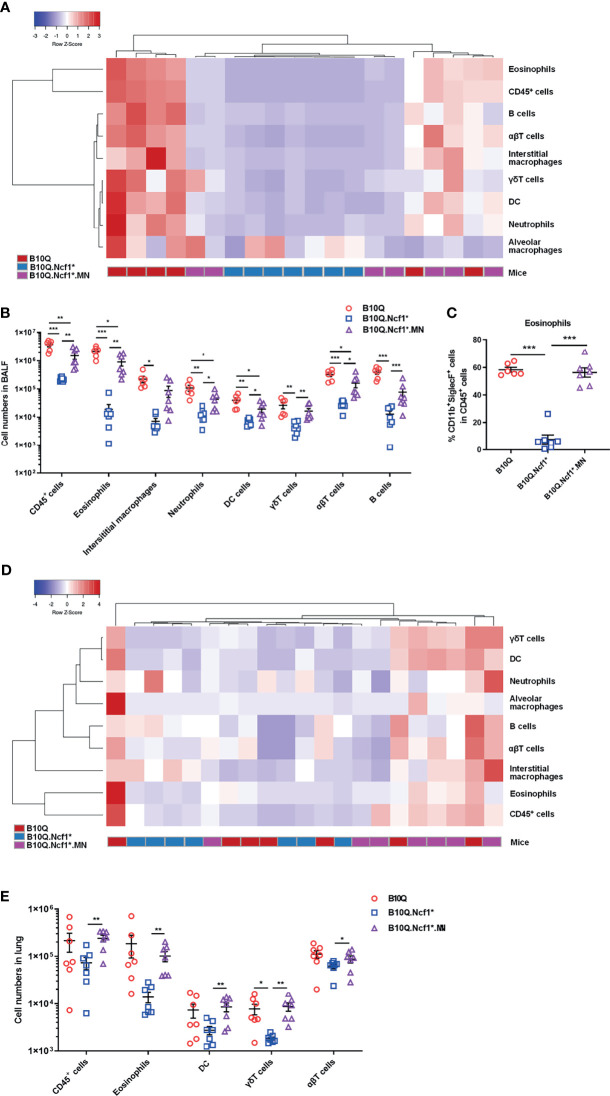
*Ncf1* deficiency reduces inflammatory infiltration in lung inflammation. **(A, B)** B10Q, B10Q.*Ncf1* ^*^ and B10Q.*Ncf1* ^*^.MN mice were induced AIPI, the number of cells in BALF were detected by flow cytometry (n = 6-7 per group). Heat map with cell profile of each mouse, and the statistical analysis of cells with differences between groups are shown. **(C)** The proportion of eosinophils (CD11b^+^SiglecF^+^ cells) in CD45^+^ cells of BALF is shown in histogram. **(D, E)** Additionally, immune cells in lung tissues (upper left lobes) from the three strains were also detected by flow cytometry (n = 7 per group). Heat map with cell profile of each mouse, and the statistical analysis of cells with difference among groups are shown. All values are expressed as means ± SEM. *P < 0.05, **P < 0.01 and ***P < 0.001, using one‐way ANOVA with *post‐hoc* comparison (Fisher’s) test.

The inflammatory cell profiles of cells infiltrating in lungs were studied by flow cytometry. The results of staining of BALF cells are visually represented *via* a cluster heat map, which revealed a clustering of the inflammatory profiles of *Ncf1* deficient mice versus the wild-type mice, and with the MN mice in between (**Figure 3A**). When *Ncf1* was deficient, the total number of cells and various inflammatory cells in BALF were significantly decreased compared with wild-type (**Figure 3B**), and the proportion of eosinophils was also decreased (**Figure 3C**). Clearly, the replenishment of *Ncf1* in alveolar macrophages could compensate for the above differences to a certain extent (**Figures 3B, C**). In addition, the population of immune cells in lung tissue was also analyzed, and the result of lung tissue was very similar to that of BALF, with reduced numbers of total immune cells and eosinophils in *Ncf1* deficient mice (**Figures 3D, E**). Taken together, the results suggest that expression of a functional *Ncf1* allows the development of lung inflammation, which is mainly depended on *Ncf1* expressing alveolar macrophages.

### *Ncf1* Alters Type 1 and 2 Responses in Lungs of AIPI

**Figure 4:**
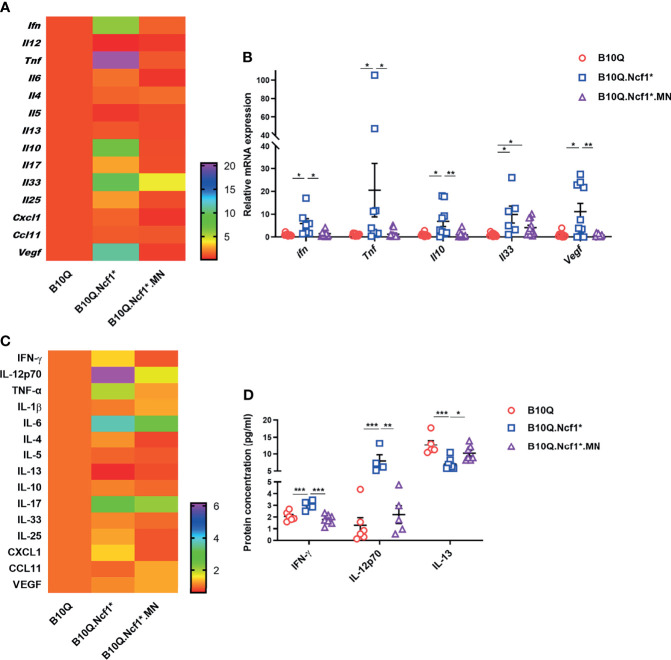
*Ncf1* alters type 1 and 2 responses in lungs of AIPI. **(A, B)** mRNA expression of cytokines and chemokines in lung tissues of AIPI mice were detected by RT-qPCR (n = 6-12 per group). **(C, D)** The protein concentration of various cytokines and chemokines in serum of three genotypes of mice were detected by Luminex assay (n = 4-7 per group). The heat maps **(A, C)** showed the mean values of gene/protein expression in each group of mice (B10.Q group is set as one in heat maps), and the statistically different gene/protein expression are shown in the histograms **(B, D)**. All values are expressed as means ± SEM. *P < 0.05, **P < 0.01 and ***P < 0.001, using one‐way ANOVA with post‐hoc comparison (Fisher’s) test.

To address the mechanism whereby *Ncf1* regulated airway inflammation, we determined the cytokine profiles of lung tissue and serum. The mRNA expression of cytokines and chemokines in lung tissue was detected by RT-qPCR (**Figures 4A, B**), and their protein concentrations in serum were detected by Luminex assay (**Figures 4C, D**). The results showed that the mRNA expression of *Ifng* and *Tnfa* in lung tissue, and the protein concentrations of IFN-γ and IL-12 in serum were increased in B10Q.*Ncf1* ^*^ mice, as compared with the wild-type mice, and their expression was decreased in MN mice (**Figures 4B, D**). In addition, the mRNA expression of *Il10*, *Il33*, and *Vegf* in lung was increased, whereas the protein concentration of IL-13 in serum was decreased, in *Ncf1* deficient mice, while MN reversed these changes to some extent (**Figures 4B, D**). Overall, *Ncf1* deficiency was associated with an enhanced Th1-type of cytokine response, an effect that could be completely neutralized by expression of *Ncf1* in macrophages. It raises the possibility that activation of the NOX2 complex in macrophages enable these cells to suppress a Th1 type of inflammatory response and thereby allowing a pathogenic Th2 type of reaction, explaining why expression of *Ncf1* in macrophages promotes an allergic type of inflammation in AIPI.

### IL-33 Induced Lung Inflammation Is Regulated by *Ncf1* Independent of Macrophages

**Figure 5:**
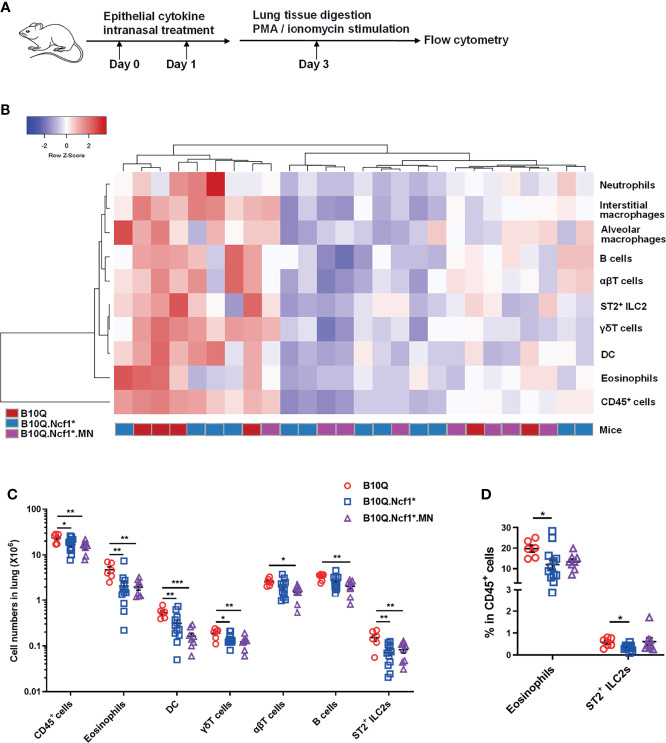
IL-33 induced lung inflammation is regulated by *Ncf1* independent on macrophages. **(A)** IL-33 was intranasally administered to mice to induce lung inflammation (n = 6-12 per group). **(B, C)** The numbers of immune cells in the whole lung tissues were analyzed by flow cytometry, and heat map with cell profile of each mouse, and the statistical analysis of cells with difference among groups are shown. **(D)** The proportion of eosinophils (CD11b^+^SiglecF^+^ cells) and ILC2 (ST2^+^Lineage^-^ cells) in CD45^+^ cells of lung are shown in histogram. All values are expressed as means ± SEM. *P < 0.05, **P < 0.01 and ***P < 0.001, using one‐way ANOVA with post‐hoc comparison (Fisher’s) test.

It was interesting to note that IL-33 and IL-25 expression was increased or showed an increased trend in *Ncf1* deficient mice, suggesting that *Ncf1* might regulate epithelial derived cytokines. These epithelial cytokines are believed to play important roles in mediating immune response in lung, and intranasal treatment of mice with IL-33 or IL-25, has been shown to induce pulmonary inflammation (25). To more directly test the role of these cytokines we treated the mice intranasally with IL-33 or IL-25 (**Figure 5A**), and the immune cell populations in lung tissues were analyzed by flow cytometry. In the IL-33 induced model, unlike the AIPI model, the heap map revealed a similarity of the inflammatory profiles between *Ncf1* deficient mice and MN mice (**Figure 5B**). The numbers of the total immune cells, eosinophils, T cells, B cells, DC, γδT cells and ILC2, were all decreased in the lungs of B10Q.*Ncf1* ^*^ mice (**Figure 5C**). In particular, the proportions of eosinophils and ILC2 were decreased due to *Ncf1* deficiency (**Figure 5D**). However, MN did not reverse the protective effect of *Ncf1* deficiency, suggesting that *Ncf1* was involved in this process through other types of cells.

On the contrary, *Ncf1* appeared to play only a minor role in IL-25 stimulated lung inflammation. The total number of inflammatory cells, or the number of eosinophils and ILC2, as well as their proportion, did not change with *Ncf1* deficiency, although macrophages, DC and γδT cells were slightly downregulated (**Figures S4A, B**). Our results suggest that only the lung inflammatory response induced by IL-33, but not IL-25, is regulated by *Ncf1*.

### *Ncf1* Deficiency Enhances Th1 Response and Inhibits ILC2 Activation in IL-33 Induced Lung Inflammation

**Figure 6:**
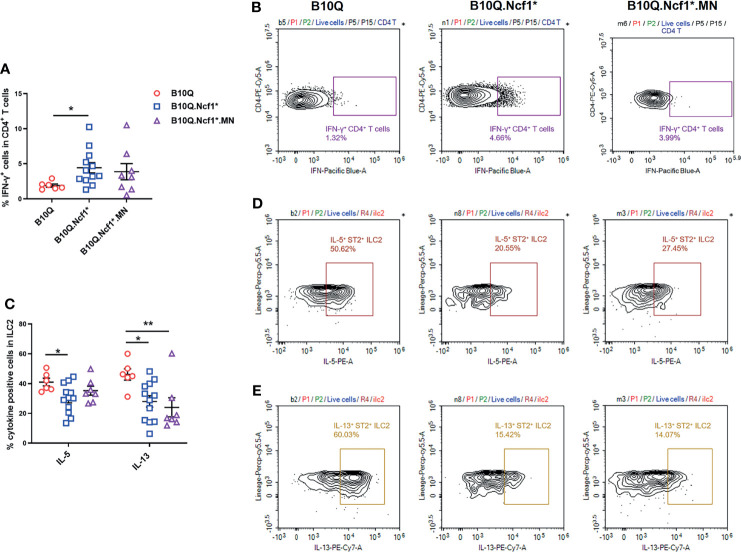
Activation of Th1 cells and ILC2 is regulated by *Ncf1* in lung inflammation induced by IL-33. The IFN-γ positive cells in CD4^+^ T cells **(A, B)**, and the IL-5 and IL-13 positive cells in ILC2 **(C–E)** were detected by flow cytometry in the whole lung tissues of mice treated with IL-33 (n = 6-12 per group). The proportion of IFN-γ positive cells in CD4^+^ T cells, and that of IL-5 and IL-13 positive cells in ILC2, were analyzed in histograms, and the representative plots are shown. All values are expressed as means ± SEM. *P < 0.05 and **P < 0.01, using one‐way ANOVA with post‐hoc comparison (Fisher’s) test.

To explain how *Ncf1* regulated IL-33 induced lung inflammation, we determined the cytokine production in this model. Since we found that *Ncf1* deficiency could enhance Th1 response in the AIPI model, and αβT cells were also downregulated in the IL-33 induced model, we analyzed the IFN-γ production in CD4^+^ T cells in the lungs by flow cytometry. The results showed that the proportion of IFN-γ^+^ cells in CD4^+^ T cells was increased in B10Q.*Ncf1* ^*^ mice compared with B10Q mice (**Figures 6A, B**). This result again underlined the importance of an enhanced Th1 response in the inflamed lungs regulated by *Ncf1*.

Next, we addressed whether ILC2 in the lung, known to be activated by IL-33, could be regulated by *Ncf1*. Therefore, the activation of ILC2 was evaluated through determining the IL-5 and IL-13 production. The results showed that the proportions of IL-5^+^ and IL-13^+^ cells within the ILC2 population, were decreased in B10Q.*Ncf1* ^*^ mice as compared with their wild-type littermates, suggesting that *Ncf1* deficiency deactivated ILC2, which could contribute to the disease protection (**Figures 6C–E**). Expression of *Ncf1* in alveolar macrophages (i.e. in the MN mouse) did not influence the decreased number of IL5^+^ and IL13^+^ ILC2 lymphocytes (**Figures 6C–E**). Activation of ILC2 was also observed in the IL-25 induced model, but in this model no difference on cytokine production in CD4^+^ T cells and ILC2 could be observed (**Figures S4C, D**).

### IFN-γ Induced Th1 Response Inhibits ILC2 Activation and Results in the Protective Effect of *Ncf1* Deficiency

**Figure 7:**
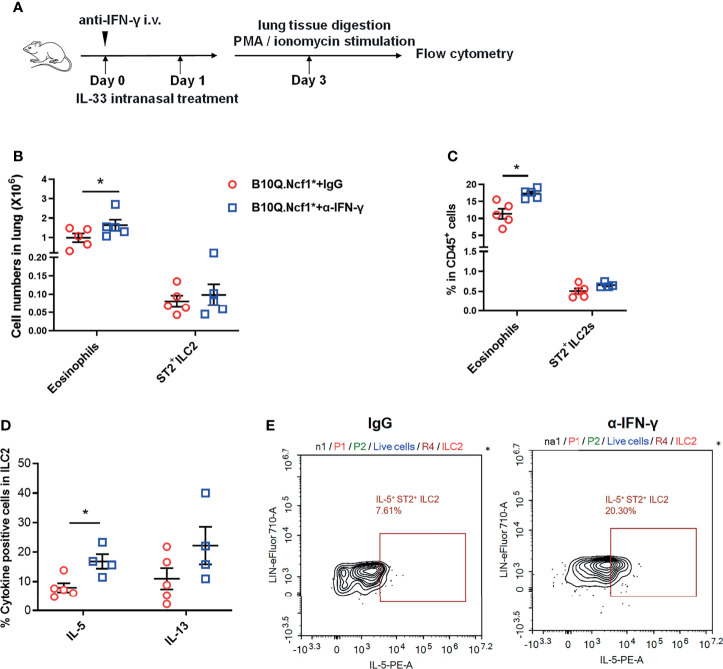
Intervention of IFN-γ rescues ILC2 activation and lung inflammation. **(A)** Anti-IFN-γ neutralizing antibodies or isotype control antibodies were used for treatment of B10Q.*Ncf1* ^*^ mice in the IL-33 treated model (n=5 per group). **(B, C)** The numbers of eosinophils and ILC2 in the whole lung tissue were analyzed by flow cytometry. The proportion of eosinophils and ILC2 in CD45^+^ cells were determined in histogram. **(D, E)** In addition, the IL-5 and IL-13 positive cells within the ILC2 population were also detected by flow cytometry of cells from the whole lung tissues of the mice. The proportion of IL-5 and IL-13 positive cells in ILC2 were analyzed in histograms, and the representative plots of IL-5 are shown. All values are expressed as means ± SEM. *P < 0.05, using student’s *t* test.

**Figure 8:**
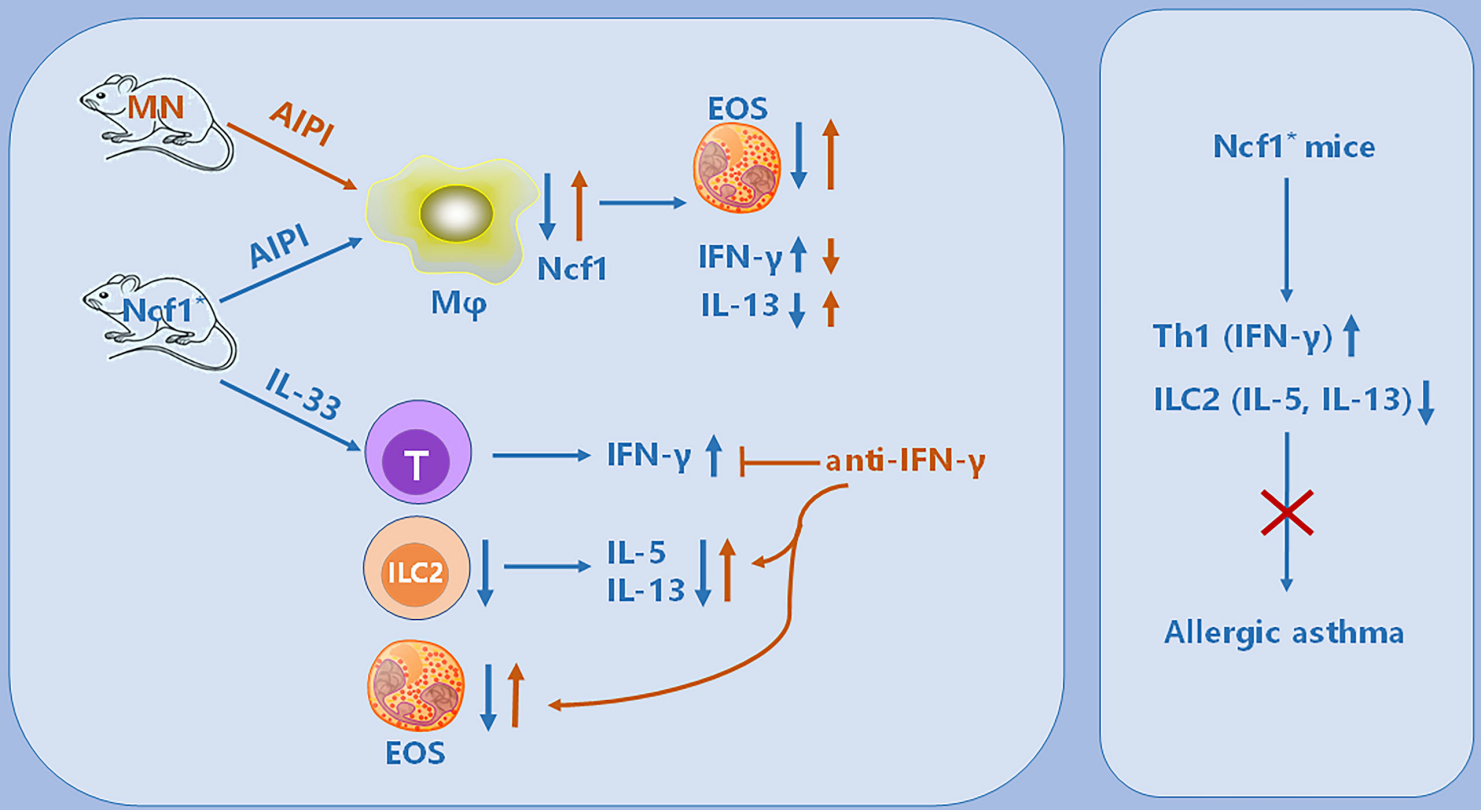
Illustration of the immunoregulatory role of *Ncf1* in pulmonary inflammation. In the OVA-induced AIPI model, *Ncf1* deficient mice were protected from lung inflammation, with reduced eosinophil (EOS) infiltration, decreased IL-13 but increased IFN-γ production. The MN mice, with alveolar macrophage-specific *Ncf1* restoration, showed the reversed inflammation severity, suggesting a residential macrophage-dependent immunoregulatory effect of *Ncf1* in the lung inflammation. In addition, *Ncf1* deficient mice stimulated with IL-33 showed reduced eosinophilic inflammation in the lung, with decreased ILC2 activation and increased IFN-γ production, while anti- IFN-γ antibody treatment to mice reversed disease severity and increased ILC2 activation. Our results indicate that *Ncf1* deficiency enhanced Th1 response and deactivate ILC2, to protect against lung inflammation in mice.

Since both Th1 enhancement and ILC2 deactivation were observed in *Ncf1* deficient mice, to reveal the causality, we blocked the IFN-γ activity in the IL-33 induced model. This was done by treating B10Q.*Ncf1* ^*^ mice with anti-IFN-γ neutralizing antibodies (**Figure 7A**). Compared with isotype IgG group, mice treated with anti-IFN-γ antibodies showed increased eosinophil numbers and proportion in the lungs, although there was no change of the ILC2 numbers (**Figures 7B, C**). The mice treated with anti-IFN-γ antibodies showed an increased proportion of IL-5^+^ ILC2, and the proportion of IL-13^+^ cells in ILC2 also showed an increased trend, suggesting that blocking IFN-γ pathway rescued the ILC2 activation (**Figures 7D, E**). Thus, our results indicate that *Ncf1* deficiency enhances Th1 response, and thereby deactivates ILC2 lymphocytes, limiting the development of lung inflammation (**Figure 8**).

## Discussion

When phagocytic and antigen presenting cells are exposed to inflammatory stimuli, the Ncf1 protein is phosphorylated and together with other proteins forms the NOX2 complex in membranes, which is the critical oxidase mediating the oxidative burst (26, 27). A deficiency of the *Ncf1* gene was first identified to be one of the genetic causative factors for development of chronic granulomatous disease (CGD) (28). Later naturally selected polymorphic alleles downsizing the ROS response, were detected, and found to be associated with several chronic autoimmune diseases in animal models and humans, such as SLE and RA (12, 14). However, the role of *Ncf1* in allergic asthma remains unclear. We therefore tested the effect of a *Ncf1* mutation, prohibiting a functional ROS response, using the classic antigen induced pulmonary inflammation, often used as a mouse model for human asthma. Our results indicate that a fully functional *Ncf1* is needed to allow the induction of AIPI, since *Ncf1* deficient mice showed a reduced immune cell infiltration in alveoli and airways. Moreover, by expressing *Ncf1* in alveolar macrophages, the protective effect of *Ncf1* deficiency was reversed.

The involvement of *Ncf1* in inflammatory diseases in several studies suggests it to play a key role as an immune regulator. It was found that *Ncf1* dependent NAPDH oxidase activity in macrophages was important for the induction of Tregs to control T cell-mediated inflammation (29), and *Ncf1* is essential for Treg-mediated CD4^+^ effector T cell inhibition (30). Interestingly, these effects are mediated by ROS derived from the antigen presenting cells, which will oxidize the T cells in the vicinity, most likely during antigen presentation (31). *Ncf1* was found to play an important role in virus induced hyperinflammatory lung injury *via* toll-like receptor (TLR) 4 pathway (18). In addition, mice lacking *Ncf1* exhibited the reduced growth of implanted melanoma and lung carcinoma tumors, and the protection on the melanoma might be caused by increased Th1 cytokines but the reduced lung carcinoma might be associated with increased Th2 cytokine production, suggesting the diverse immunoregulatory effects of *Ncf1* in different disease conditions (15). In the present study, elevated levels of type 1 cytokines including IFN-γ and IL-12 and decreased level of type 2 cytokine IL-13 in lung or serum of the AIPI model, suggested that allergic inflammation might be regulated by *Ncf1* through reprogramming the type 1 and type 2 immune homeostasis, which is an important supplement to the immune regulation mechanism of *Ncf1* in lung inflammation.

Recent studies have highlighted an important role of epithelial cytokines, including IL-25, IL-33 and TSLP, in lung inflammation. We found that the expression of IL-33 and IL-25 was increased in *Ncf1* deficient mice, suggesting that they might be involved in the *Ncf1*-mediated regulation of pulmonary inflammation. IL-33, binding to its receptor ST2 on mast cells, Th2 cells, and ILC2, can directly induce airway inflammation and play an important role in the activation and differentiation of Th2 cells (32–34). IL-25 is rapidly released in the airway in response to allergenic stimuli, contributing to allergic inflammation, causing eosinophilia, inducing Th2-biased inflammation and excessive production of IL-4, IL-5 and IL-13 (35). Importantly, both IL-33 and IL-25 could activate ILC2, leading to production of IL-5 and IL-13, which mediates lung inflammation (25). Stimulation with either IL-33 or IL-25 in mice can induce lung inflammation, which are now widely used as models for studies of ILC2 regulation of lung inflammation (36, 37). An IL-33 connected response has been shown to mediate severe arthritis in mice with the *Ncf1* mutation, provided that the immune response is directed towards Col2 in joint cartilage (38), which gave us some clues related to IL-33 and *Ncf1*. We therefore induced IL-33 and IL-25 treated mouse models using *Ncf1* deficient mice. In line with the previous finding, *Ncf1* deficiency reduced eosinophil infiltration, and increased IFN-γ production in CD4^+^ T cells, re-emphasizing the importance of Th1 response enhancement in the inflammatory regulation of *Ncf1*. Besides, *Ncf1* deficiency also inhibited the activation of ILC2, and decreased IL-5 and IL-13 production. However, supplementing macrophage *Ncf1* could not reverse the above effects, suggesting a contributing effect by other cell types expressing *Ncf1*. In addition, it was found that the regulation and activation of ILC2 by IL-33, as compared with IL-25, might be different (39, 40). We also found that *Ncf1* deficiency had limited effect on the IL-25 induced model, indicating that IL-33 but not IL-25 is more closely associated with the regulatory effect by *Ncf1*.

It is clearly of interest to reveal the causality of Th1 enhancement and ILC2 deactivation in the *Ncf1* deficient context, which could be helpful for understanding the mechanism of the protective effect of *Ncf1* deficiency. It has been reported that IFN-γ production, induced through TLR9 activation, could alleviate ILC2-driven airway hyperresponsiveness (AHR) and airway inflammation (41). IFN-γ could inhibit ILC2 expansion and IL-13 expression, thereby attenuating rhinovirus-induced mucous metaplasia (42). Thus, we thought that the antagonistic function of IFN-γ on ILC2 activation could provide a clue to our question, leading us to an experiment to block IFN-γ. We applied anti-IFN-γ antibody to IL-33 treated *Ncf1* deficient mice, which rescued ILC2 activation and reversed the disease remission to some extent, suggesting that *Ncf1* deficiency enhanced the Th1 response, thereby deactivating ILC2, to protect mice from lung inflammation.

It is interesting that the enhancement of the Th1 response due to *Ncf1* deficiency contributes to regulate the type 2 allergic inflammation, since a stronger Th1 response seems to be closely related to the disease-promoting effect of *Ncf1* in autoimmune diseases. The Th1 response, as well as the Th17 response, regulated by *Ncf1* deficiency, may be important reasons for mice to be more susceptibility to autoimmune arthritis or lupus. NADPH oxidase deficiency could regulate Th lineage commitment to modulate autoimmunity, and superoxide provides a third signal for CD4 T cell effector responses (43, 44). ROS derived from the NOX2 complex on APCs, most likely hydrogen peroxide, can act as an immunological transmitter onto T cells and affect T cell signalling (31). However, the exact molecular mechanism remains unclear, which needs additional studies.

At present stage, our study has some limitations. We used OVA or epithelial cytokines to induce airway inflammation in this study. Although they all share many characteristics with asthma, house dust mite (HDM) induced model might be a better one to mimic the occurrence of human asthma from the induction mechanism. The role of *Ncf1* in HDM animal model and human asthma will be our prior focus in the future. In addition, we now mainly focused on the regulatory effect of *Ncf1* on lung inflammation from the aspects of histology and cytology. Due to the limitation of experimental conditions, we did not analyze the lung function, limiting us to fully understand the role of *Ncf1* in asthma pathogenesis. However, the regulatory effect of *Ncf1* on Th1 and Th2 in pulmonary inflammation should be convincing, and we also hope to further confirm the effect of *Ncf1* on AHR in the future.

In conclusion, we confirm *Ncf1* as an important immune regulator in pulmonary inflammation, orchestrating type 1 and 2 immune responses. This study is helpful for us to understand the pathogenesis of asthma and provides a potential target for human asthma. *Ncf1* and its mediated oxidative stress may be a candidate intervention pathway in the treatment of pulmonary allergic inflammation.

## Data Availability Statement

The original contributions presented in the study are included in the article/**Supplementary Material**. Further inquiries can be directed to the corresponding authors.

## Ethics Statement

The animal study was reviewed and approved by the Institutional Animal Ethics Committee of Xi’an Jiaotong University.

## Author Contributions

All authors contributed to the discussion, writing, and revising of the manuscript. ML designed the study, performed the majority of the experiments, analyzed the data and wrote the manuscript. WZhang, JZ, and XL performed experiments and analyzed data. FZ provided expert technical assistance. WZhu, LM, RH, and SL designed the research, analyzed the data, revised the manuscript, supervised and took the overall responsibility of the study. All authors contributed to the article and approved the submitted version.

## Funding

This work is supported by the National Natural Science Foundation of China (81970029, 82171724 and 82171784), the Shaanxi Province Natural Science Foundation (2020JQ082), and grants from the Swedish Strategic Science Foundation (SSF), the Knut and Alice Wallenberg Foundation, and the Swedish Research Council.

## Conflict of Interest

The authors declare that the research was conducted in the absence of any commercial or financial relationships that could be construed as a potential conflict of interest.

## Publisher’s Note

All claims expressed in this article are solely those of the authors and do not necessarily represent those of their affiliated organizations, or those of the publisher, the editors and the reviewers. Any product that may be evaluated in this article, or claim that may be made by its manufacturer, is not guaranteed or endorsed by the publisher.
