# Supplementary material for: Ncf1 Governs Immune Niches in the Lung to Mediate Pulmonary Inflammation in Mice
Source: Front Immunol. 2021 Dec 14;12:783944. doi: 10.3389/fimmu.2021.783944 (PMC8712564; doi:10.3389/fimmu.2021.783944)
Supplement: Supplementary file 1 [file DataSheet_1.docx]

**Supplementary table 1. Sequence information for primers**

| **Gene name** | **Sequence (from 5’ to 3’)** |
| --- | --- |
| ***Il4*** | F: CCA TAT CCA CGG ATG CGA CA |
|  | R: AAG CAC CTT GGA AGC CCT AC |
| ***Il5*** | F: AGC AAT GAG ACG ATG AGG CT |
|  | R: AGC ATT TCC ACA GTA CCC CC |
| ***Il10*** | F: AGG CGC TGT CAT CGA TTT CT |
|  | R: ATG GCC TTG TAG ACA CCT TGG |
| ***Il13*** | F: CCA TCT ACA GGA CCC AGA GGA |
|  | R: CTC CCC AGC AAA GTC TGA TGT |
| ***Ifng*** | F: AAGACAATCAGGCCATCAGCA |
|  | R: CTCATTGAATGCTTGGCGCT |
| ***Il12*** | F: TTCTCACCGTGCACATCCAA |
|  | R: GAGGAGGTAGCGTGATTGACA |
| ***Tnfa*** | F: TCAGCCTCTTCTCATTCCTGC |
|  | R: TTGGTGGTTTGCTACGACGTG |
| ***Il6*** | F: AAGAAAGACAAAGCCAGAGTC |
|  | R: CACAAACTGATATGCTTAGGC |
| ***Il17*** | F: ACTACCTCAACCGTTCCACG |
|  | R: TTCCCTCCGCATTGACACAG |
| ***Il33*** | F: AGCATTTGCTGCGTCTGTTG |
|  | R: GACTTGCAGGACAGGGAGAC |
| ***Il25*** | F: GAAGTGGAGCTCTGCATCTGT |
|  | R: CCGATTCAAGTCCCTGTCCAA |
| ***Cxcl1*** | F: ACTCAAGAATGGTCGCGAGG |
|  | R: GTGCCATCAGAGCAGTCTGT |
| ***Ccl11*** | F: GCTCCACAGCGCTTCTATTC |
|  | R: GCTTTCAGGGTGCATCTGTTG |
| ***Vegf*** | F: CGGGCCTCGGTTCCAG |
|  | R: CTGCTCTCCTTCTGTCGTGG |
| ***β-actin*** | F: CAC TGT CGA GTC GCG TCC |
|  | R: TCA TCC ATG GCG AAC TGG TG |

**Supplementary table 2. Antibody information used for flow cytometry**

| **Antibody** | **Clone No.** | **Manufacturer** | **Cat. No.** |
| --- | --- | --- | --- |
| BV785 anti-mouse CD45 | 104 | BioLegend | 109839 |
| APC anti-mouse/human CD11b | M1/70 | BioLegend | 101212 |
| APC-Cy7 anti-mouse CD11c | N418 | BioLegend | 117324 |
| FITC anti-mouse F4/80 | BM8 | BioLegend | 123108 |
| AF700 anti-mouse TCRβ | H57-597 | BioLegend | 109224 |
| PE anti-mouse γdTCR | GL3 | Becton Dickinson | 553178 |
| PE-Cy7 anti-mouse B220 | RA3-6B2 | BioLegend | 103222 |
| Pacific blue anti-mouse Ly6G | 1A8 | BioLegend | 127612 |
| PerCP-Cy5.5 anti-mouse SiglecF | E50-2440 | Becton Dickinson | 565526 |
| PE-Cy5 anti-mouse CD4 | RM4-5 | BioLegend | 100514 |
| BV421 anti-mouse ST2 | U29-93 | Becton Dickinson | 566309 |
| Pacific blue anti-mouse IFNγ | XMG1.2 | BioLegend | 505818 |
| PE anti-mouse/human IL-5 | TRFK5 | Becton Dickinson | 554395 |
| PE-Cy7 anti-mouse IL-13 | eBio13A | eBioscience | 25-7133-82 |
| Pacific blue anti-mouse CD68 | FA-11 | BioLegend | 137028 |
| PerCP-Cy5.5 mouse lineage antibody cocktail | 145-2C11, M1/70, RA3-6B2, TER-119, RB6-8C5 | Becton Dickinson | 561317 |

**Supplementary figure 1.**

**Supplementary figure 1. The gating strategy of immune cells using flow cytometry. (A)** Gating CD45^+^ cells, alveolar macrophages, neutrophils, eosinophils, interstitial macrophages, DC, γδT cells, αβT cells and B cells in lung tissues and BALF cells. **(B)** Gating CD4^+^ T cells and IFN-γ expressing cells in lung tissues. **(C)** Gating ST2^+^Lineage^-^ ILC2, and IL-5 and IL-13 expressing cells in lung tissues.

**Supplementary figure 2.**

**Supplementary figure 2. Fluorescence-minus-one (FMO) controls of all antibodies used in flow cytometry.**

**Supplementary figure 3.**

**Supplementary figure 3. Comparison of the lung histology and immune cell profile of lung tissues between naïve *Ncf1* deficient mice and wildtype mice. (A)** Lung tissues of naïve B10Q and B10Q.*Ncf1*^*^ mice were stained by H&E. Bar=100 μm. **(B-C)** The number of immune cells in lung tissues and BALF of naïve B10Q and B10Q.*Ncf1*^*^ mice were detected by flow cytometry (n = 6 per group). All values are expressed as means ± SEM.

**Supplementary figure 4.**

**Supplementary figure 4. IL-25 induced lung inflammation was not regulated by *Ncf1*.** IL-25 was intranasally administered to mice to induce lung inflammation (n = 5 per group). **(A-B)** The number and proportion of inflammatory cells were detected by flow cytometry. **(C-D)** The IFN-γ positive cells in CD4^+^ T cells, and the IL-5 and IL-13 positive cells in ILC2 were detected. All values are expressed as means ± SEM. **P* < 0.05 and ***P* < 0.01, using one‐way ANOVA with post‐hoc comparison (Fisher’s) test.
